# Staff Nurses’ Perceptions and Experiences about Structural Empowerment: A Qualitative Phenomenological Study

**DOI:** 10.1371/journal.pone.0152654

**Published:** 2016-04-01

**Authors:** Peter Van Bogaert, Lieve Peremans, Nadine Diltour, Danny Van heusden, Tinne Dilles, Bart Van Rompaey, Donna Sullivan Havens

**Affiliations:** 1 Nursing and Midwifery Sciences, Centre for Research and Innovation in Care, Faculty of Medicine and Health Sciences, University of Antwerp, Antwerp, Belgium; 2 Nursing Department, Antwerp University Hospital, Antwerp, Belgium; 3 School of Nursing, The University of North Carolina at Chapel Hill, Chapel Hill, NC, United States of America; 4 Department of Primary and Interdisciplinary Care, University of Antwerp, Antwerp, Belgium; 5 Mental Health and Wellbeing Research Group, Vrije Universiteit Brussel, Brussels, Belgium; University of Stirling, UNITED KINGDOM

## Abstract

The aim of the study reported in this article was to investigate staff nurses’ perceptions and experiences about structural empowerment and perceptions regarding the extent to which structural empowerment supports safe quality patient care. To address the complex needs of patients, staff nurse involvement in clinical and organizational decision-making processes within interdisciplinary care settings is crucial. A qualitative study was conducted using individual semi-structured interviews of 11 staff nurses assigned to medical or surgical units in a 600-bed university hospital in Belgium. During the study period, the hospital was going through an organizational transformation process to move from a classic hierarchical and departmental organizational structure to one that was flat and interdisciplinary. Staff nurses reported experiencing structural empowerment and they were willing to be involved in decision-making processes primarily about patient care within the context of their practice unit. However, participants were not always fully aware of the challenges and the effect of empowerment on their daily practice, the quality of care and patient safety. Ongoing hospital change initiatives supported staff nurses’ involvement in decision-making processes for certain matters but for some decisions, a classic hierarchical and departmental process still remained. Nurses perceived relatively high work demands and at times viewed empowerment as presenting additional. Staff nurses recognized the opportunities structural empowerment provided within their daily practice. Nurse managers and unit climate were seen as crucial for success while lack of time and perceived work demands were viewed as barriers to empowerment.

## Introduction

Continuous changing markets and consumers within a global economy need adaptive organizations. Adaptive organizations systematically implement worker involvement and decision-making processes in order to be more productive, resulting in engaged employees and excellent outcomes [1]. Kanter [2] described workplace social structures that enable employees to mobilize human and material resources to accomplish meaningful work through access to the necessary information, support and resources in the work setting. According to Kanter, sources of empowerment will determine the extent to which employees have developed an organizational network of alliances (e.g. development of informal power) and jobs that have a large degree of discretion, are visible and important to organizational goals (e.g. having formal power). Kanter’s theoretical framework defines structural empowerment as the following work characteristics; formal and informal power; access to information; opportunities to learn and personal development; and supportive relationships (e.g. superiors, peers and subordinates). Furthermore, psychological empowerment is the response to certain empowering conditions and an outcome of structural empowerment [3]. Spreitzer, operationalized psychological empowerment as the psychological state of employees who experience a certain extent of *meaning*, *competence*, *self-determination* and *impact* in their job [4]. Based on a longitudinal study of 185 randomly selected nurses, Laschinger et al. [5] reported that changes in perceived practice environment based on structural empowerment were associated with positive changes in psychological empowerment and job satisfaction. Later on Laschinger et al. [6] reported that aspects of structural empowerment (e.g. access to information and resources; the extent of support and formal power) strongly influenced work engagement, which subsequently affected nurse-reported work effectiveness (e.g. the extent to which nurses felt empowered to work effectively in their current environment).

A large body of knowledge reveals associations between balanced, healthy and supportive nurse practice environments and favorable nurse outcomes such as low burnout, low turnover intentions, high work engagement, and job satisfaction [7–8–9–10–11–12–13]. In addition, these practice environments are associated with favorable patient outcomes including relatively low mortality and co-morbidity rates and adverse events [14–15–16–17].

Our previous research findings derived from a cross sectional survey of 1,200 registered from two independent acute care hospitals and one acute care hospital group with six hospitals [18] in Belgium demonstrated positive associations between characteristics of empowerment (e.g., balanced workload; decision latitude; and social capital), and various outcome variables such as low feelings of burnout; low turnover intentions; nurse job satisfaction; and favorable nurse-assessed quality of care. In addition, workload, decision latitude and social capital were influenced by the quality of the practice environment (e.g. nurse-physician relationships, nurse management at the unit level as well as hospital management & organizational support). Decision latitude was described as the ability to make decisions, be creative, and use and develop professional and personal skills at the workplace. Furthermore, social capital was described as shared values and perceived mutual trust within teams. Associations were shown through a carefully prepared and tested model (e.g. structural equation modelling) based on previous studies [19–20]. Further analyses showed associations between study variables at the nursing unit level [21] that underpinned nurses’ shared experiences within their units and the importance of nursing teams. In one of our study hospitals (600-bed university hospital) longitudinal survey research based on three measurement periods (2006, 2011, 2013) using the same study variables was conducted. The study investigated the effect of transforming the hospital organization from a classic hierarchical and departmental structure (see Method section; Study context) to a flat and interdisciplinary model [22]. During the study period, nurses practicing on 21 clinical units reported improvement in the nurse practice environment associated with improved outcome variables. These findings demonstrated the importance of nurse practice environments characterized by good interdisciplinary relationships as well as support from supervisors, peers, nurse administrators and executives within a structure of shared values and shared governance (e.g. structural empowerment). Moreover, nurses were able to practice more professionally focusing on quality patient care. To better understand the evolution the study hospital was going through as well as the role of structural empowerment in supporting a favorable nurse practice environment leading to better patient outcomes, a qualitative study was conducted. The aim of the study reported in this article was to investigate staff nurses’ perception and experiences about structural empowerment and perceptions regarding the extent to which structural empowerment supports safe quality patient care.

## Methods

### Design

We used a phenomenological method within a constructivist paradigm, from the staff nurse perspective. We aimed to reveal essential general meaning and structures about a phenomenon. One investigator performed individual semi-structured interviews, a technique appropriate to the phenomenological approach.

### Study context

To more accurately address complex patient needs within a continuously evolving medical science and socioeconomic context, the study hospital’s management decided in 2007 to transform the hospital organization [22]. The aim of the transformation process was to develop a flat and interdisciplinary organizational structure from what was a classic hierarchical and departmental structure. The overall goal was that nurses and other healthcare workers would be more involved and empowered to adapt daily practice to meet evolving patient needs in order to achieve best care. Inspired by the American Nurses Credentialing Centre’s (ANCC) Magnet Hospital concept [23–24] and previous studies [25–26–27–28] three of the original 14 *forces of magnetism* guided the hospital transformation to achieve and support better nursing performance: (1) flat organizational structures, where unit-based decision making prevailed, with sufficient nurse representation in the organizational committee structure; (2) a participative management style incorporating sufficient feedback from staff nurses and the presence of visible and accessible nursing leaders; and (3) positive interdisciplinary relations with mutual respect amongst all disciplines. Several initiatives to support structural empowerment based on these three principles were implemented (Table 1).

**Table 1:** Hospital initiatives to promote structural empowerment (Most relevant initiatives, not exhaustive).

Initiatives	Outset	Content	Implementation strategy
Structural support for clinical units	2009	Quarterly unit meetings; agenda based on team member consultation; annual unit goal setting; meetings between staff nurses and physicians about dedicated unit level topics	Policy development; implementation process based on meetings with CNO and nursing unit managers
Nursing councils	2008	Programs for the transition process of newly introduced staff nurses developed by nurse preceptors	Setting goals, strategies and implementation of programs; training programs for nurse preceptors
	2010	Programs to enhance patient safety and infection control in nursing practice and clinical units developed by dedicated champions	Setting goals, strategies and implementation of programs; training programs for champions

To operationalize the described goals, in 2012 the hospital introduced the Productive Ward–Releasing Time to Care**™**. The aim of this program was to support improvements in clinical unit care delivery within the structure of the hospital transformation process. Productive Ward–Releasing Time to Care™ also known as *the productive ward* was developed by the National Health Service (NHS) Institute for Innovation and Improvement and launched in England in 2007. The program provides fifteen modules based on *lean methodology* to systematically enhance the delivery of safe, high quality patient care [29]. These program modules support review of processes to identify and eliminate activities, which add no value to patients (i.e., eliminating waste). The program’s aim is to improve patient satisfaction due to increased provision of care by staff and subsequent improved clinical and safety outcomes. A structured implementation approach with commitment (e.g. engagement and understanding concepts) by board members, physicians, nursing administrators, managers as well as staff nurses is vital to assure sustained improvements in care delivery. The *productive ward program* supports structural empowerment through three foundation modules (see Table 2). The programme provides the tools and methods to improve the quality of care at the ward level by encouraging nurses to look at how their ward is organized; and empowering them with the tools and skills to make changes, thus allowing them to spend more time with their patients [30]. Moreover, it encourages clinical nursing teams to take ownership and control of ward-based process improvements whilst instilling a culture of measurement and improvement [31]. One tangible reported output is opportunities for the ward-based team to make structural changes in the use of the ward space and clinical environment to improve efficiency in terms of time, effort and money [32].

**Table 2:** Structural empowerment within the Productive Ward–Releasing Time to Care™.

Initiatives	Outset	Content	Implementation strategy
Foundation module—*knowing what and how we are doing*	2012	Unit vision—setting, measuring and evaluation of unit key performance indicators	
Foundation module–*the well organized ward*	2012	Unit level assessment of nurse practice environment and working conditions	Unit level training and coaching sessions; unit meetings and workshops
Foundation module—*patient status at a glance*	2013	Unit level patient status- overview of key characteristics	

Moreover, at the same time, many change initiatives were ongoing within the hospital as well as an accreditation process (Joint Commission International—JCI) as a part of hospital accountability to various stakeholders such as the government and the public (e.g. patients) during the study period. Health care accreditation is a process in which a nongovernmental entity such as JCI, which is separate and distinct from the health care organization, assesses the organization to determine if it meets a set of standards. These standards are designed to improve quality of care and yield continuous improvement in structures, processes, and outcomes [33].

### Study participants and study period

The purposive sample consisted of typical cases of staff nurses practicing on medical or surgical units of a 600-bed university hospital. We assumed that medical and surgical nursing units are relatively comparable in terms of nurse practice environments so we can expect similar perception and experiences about structural empowerment. Therefore, for this study, staff nurses from intensive care units, the operation theatre, the emergency care unit, pediatric care unit or maternal care unit were excluded. In addition, nurse aids, licensed practical nurses, midwives and nurse managers were also excluded, because of their different practice relationships with patients and/or different preparation and training.

The ethics committee of the Antwerp University Hospital Belgium approved the study. The process to select participants was based on a short list of 34 potential candidates proposed by nursing unit managers from 5 medical units and 5 surgical units. The investigator contacted each candidate through email, resulting in study participation by 8 staff nurses. The first selected nurses (snowballing) recommended three additional staff nurses and they were willing to participate resulting in 11 study participants. Participation was voluntary and the investigator informed all participants about the study (e.g. signed informed consent by participants was obtained). Data were collected until saturation was reached on the research topics. The investigator conducted the semi-structured interviews between January and March 2014.

### Procedure and data analysis

The semi-structured interviews, focused on two topics *empowerment* and *decision-making processes* with five and seven items respectively (see Table 3) were performed through a dedicated scenario (e.g. introduction, interview, ending) and each participant had to complete a short questionnaire about their demographic characteristics. All interviews were recorded and the investigator took notes during the interview.

**Table 3:** Semi-structured interview: topics and items.

Topic	Item
*Empowerment*	What does empowerment mean for you?
	What is your experience with empowerment in your practice environment?
	Which aspects influence opportunities to become empowered in your pratice environment?
	Who/what impacts opportunites to become empowered. Nurse manager? Physicians? Hospital policies?
	How does involvement in decision-making processes impact the quality of care?
*Decison-making processes*	Do decision-making processes promote quality of care and patient safety and how?
	Which decision-making processes do staff nurses influence?
	Which decision-making processes do you have influence on?
	To what extent do you wish to be involved in decision-making processes?
	What you do not wish to decide in your practice environment?
	To what degree are staff nurses involved in decisions about matters of patient care?
	Is there a possibility that empowerment can contribute to more involvement in decision-making processes?

A second investigator along with the study investigator coded one written interview independently and both investigators developed a codebook in consensus. Four interviews were coded on verbatim transcripts and the six others were coded directly through the recordings by the study investigator based on the codebook.

The thematic approach was used to conduct the analysis. Firstly we looked at the particular experiences of the participants and then we identified the different themes common to the phenomenon. Various themes and the relationships between themes were identified and described. This qualitative thematic data analysis was supported by NVivo 10 software (QRS International).

#### Rigour

Credibility was achieved through the involvement of two researchers who analyzed one written interview independently and developed a codebook in consensus and the use of verbatim quotes to provide the study participants voice along with researchers’ data interpretations, respectively [34–35]. In case of disagreement on the coding there was an additional discussion in a larger team of researchers. Self-reflection was an important issue for dependability. Researchers were stimulated to put their own ideas on paper before starting the interviews and the analyzing. This created for the researcher a constant awareness about the own background and perspective.

## Findings

Staff nurses’ demographic characteristics are summarized in Table 4.

**Table 4:** Study population demographic characteristics.

N = 11	N
Female	10
Age	
< 30 yrs	4
30–40 yrs	3
41–50 yrs	1
> 50 yrs	3
Tenure in current hospital	
<10 yrs	4
10–20 yrs	3
21–30 yrs	1
> 30 yrs	3
Full/Part time status	6
≥ 75%	4
< 75%	
Type of Nursing unit	
medical unit	4
surgical unit	7
Educational Qualifications	
Baccalaureate degree in nursing	9
Master’s degree in nursing	2
Additional training	
Management and leadership	1
Wound care	1
Additional assignment	2
Mentor	2
Member nursing council	1

The identified themes based on thematic analyses of 11 participants’ interviews about the first topic empowerment were: *meaning of empowerment*, *experiences with empowerment*, *opportunities from being empowered* and *challenges for hospital policy*. The identified themes of the second topic decision-making processes were: *level of involvement*, *involvement and quality of care*, *involvement through empowerment* and *involvement and empowerment and level of education and training*.

### Empowerment

#### Meaning of empowerment

Four study participants didn’t know the meaning of empowerment although the word sounded familiar. One participant conducted a search and another asked a colleague about the meaning of empowerment. Participants with a master’s degree or additional management and leadership training were well aware of the meaning of empowerment. Empowerment was understood as involvement in decision-making processes, support to participate in hospital internal governance and policy, ability to express your opinion, opportunities to make your voices heard and being heard. Involvement extended from opportunities related to the practice environment, patient related matters and concerns about hospital internal governance and policy.

*Empowerment means to me the extent to which I feel involved with policy decisions within our practice environment*, *common decisions about nursing … any issue … patient related to policy decisions such as JCI-accreditation (e*.*g*. *Joint Commission international accreditation) and other … (Interviewee 5)*.

Two participants described empowerment as organizational support for personal development within their practice through educational and training programs. Additional aspects included team collaboration and the power to work jointly and bottom up. In addition, empowerment connoted strength, the strength to innovate and a certain level of autonomy. One participant defined empowerment as involvement in decision-making processes related to direct patient care.

### Experiences with empowerment

All respondents experienced empowerment in their practice environment. Moreover, they mentioned aspects of structural empowerment through various hospital initiatives such as *the productive ward* and *JCI-accreditation* as being related to enhanced empowerment. Moreover, staff nurses could participate in committees, and educational and training programs. Yearly training and workshop initiatives were present, such as *eight hours for everyone*, which was seen as informative and useful. Participating in workshops about certain themes such as models of care or patient safety through incident reporting was assessed as very useful. A few mentioned the *evidence based practice* training they recently participated in and the access to scientific evidence as an initiative that promoted empowerment. Others mentioned the opportunities that evolved from training and personal development or the involvement with the development of *care indicators* on their units.

Participants felt that support within their nursing team makes nurses stronger and more resilient. Unit climate was viewed as playing a prominent role in this. Many staff nurses reported that their nursing team was most important to empowerment. Problems within nursing teams make empowerment unachievable.

*We have a close team*, *and that is most important in our daily practice regardless of hospital internal governance and policy and empowerment (Interviewee 1)*.

Unanimously, respondents reported that nursing unit managers provide opportunities for empowerment. For example, they reported that staff nurses within their nursing units had autonomy related to patient care. They also reported that nursing unit managers were supportive and served as the go between for hospital internal governance and policy. Participants noted that nursing unit managers played a crucial role for empowerment, without their support empowerment was impossible. The relationship between staff nurses and their nursing unit manager was central to experiencing empowerment.

*I feel supported by our nursing unit manager*, *I recently came to work in this unit and I can consult always with colleagues when necessary (Interviewee 2)*.

*I think it comes in the first place from our nursing unit manager*, *but at the moment it is somewhat missing*. *I think our previous nursing unit manager had supported empowerment more*, *you had with her the feeling of respect about what you are doing (Interviewee 11)*.

Opportunities to be engaged in committees (e.g. nursing department and hospital) or to have an additional role as a champion were present and promoted a certain level of autonomy and responsibility. They reported personal involvement within various projects and initiatives, they perceived that they were being heard and they reported positive feelings about their practice. Employees’ involvement was assessed as crucial. While they reported that becoming empowered presented challenges, they perceived that nurses needed to take these challenges on.

*I experience empowerment*, *yes I do*, *I am a nurse preceptor and that gives me the opportunity to be involved with nurse undergraduates’ training and coaching in our hospital*. *As a nurse preceptor I can consult skilled colleagues when necessary (Interviewee 10)*.

*Yes*, *I am a nurse preceptor and I have access to various scientific databases and that gives us the opportunity to search for scientific evidence*. *That is fine*, *however we lack time and opportunities within our daily practice (Interviewee 11)*.

Participants reported that the physicians’ role in creating empowerment was limited. It was a sensitive subject. Empowerment through physicians for the participants meant joint decision-making related to direct patient care. The relationship with physicians was experienced as open and supportive, but study participants had the feeling that physicians didn’t know much about empowerment. Participants perceived that physicians did not motivate staff nurses, had no contribution to empowerment and that they were a limited part of change initiatives.

*Physicians*, *a difficult subject*, *our physicians need to be trained about empowerment; they will be interested I think*, *but at the moment they lack knowledge and insight*. *I think that physicians still have the attitude that physicians command and nurses act*. *If empowerment means that nurses will make decisions too and take initiatives beyond nursing practice on other aspects*, *clinically as well organizationally*, *that will be a difficult case for physicians (Interviewee 6)*.

Participants who had longer tenure in the hospital experienced more empowerment when compared with participants who had not worked in the hospital as long. These nurses were more aware of the changes in the hospital and on their team than younger nurses who were newer to practice.

*We experience clearly a new approach in the hospital and in our team (Interviewee 8*.*)*.

### Decision-making processes

Staff nurses agreed to be involved with patient related care policy within their units. Hospital wide issues were more difficult to be involved in and they were generally seen as issues for nursing and hospital management.

*Joint-decision-making*? *Related to direct patient care*, *sure*, *… involvement with matters and concerns about patients*, *that will be interesting*, *… however hospital internal governance and policy … not necessarily*, *… only when relevant to nursing (Interviewee 8)*.

*I will be engaged with procedures and guidelines … I volunteered to support our wound nurse specialist to develop a home care procedure to achieve more continuity of care … an important issue in my view (Interviewee 8)*.

Staff nurses recognized the importance of standardization and uniformity of care, although they voiced certain negative feelings about the *JCI-accreditation* requirements. They were convinced that standardization and uniformity support quality of care and patient safety and provided confidence and trust in their daily care. Moreover involvement and participation also provided a sense of importance. Staff nurses having their voices heard about concerns, was essential and supportive to job satisfaction.

*What you have decided yourself*, *you will support more*, *because you know very well why (Interviewee 7)*.

Empowerment supported involvement in decision-making processes, but the effect on quality of care and patient safety was for most participants unclear. During the study period, several change initiatives were taking place in the hospital. The effect of these initiatives was unclear. Moreover, during the period of data collection, staff nurses perceived that direct patient care delivery was under pressure. However, it was clear to them that joint decision-making improved efficiency.

*I think that staff nurses stand by their patients*, *especially me; … I will always choose the best care for my patients*. *So the more I can participate to develop procedures and guidelines that support better care*, *the more I feel I am involved and engaged*. *Internal governance and policy decisions*, *… not so … they are of course all related … but I prefer more nursing care matters and concerns … (Interviewee 1)*.

Involvement in decision-making processes was highly related to nurses’ commitment. Moreover, it was not always clear about which subjects staff nurses could participate in.

They felt that systematically acquired knowledge was important to being empowered as well as critical reflection on certain matters.

In addition to clear favorable perceptions and experiences about empowerment, unfavorable aspects were also reported. Some participants were convinced that work demands were increasing because of aspects of empowerment and joint decision-making. Such additional tasks associated with empowering environments were viewed by some staff nurses as demotivating and served to deplete their dedication to participate in decision-making processes, individually as well within teams.

## Education and training

Nurses’ educational level was viewed as potentially supportive of more involvement. Younger colleagues with bachelor’s degrees were better prepared to be involved than older colleagues. Staff nurses with a master’s degree perceived that they were better trained to guide certain change initiatives and decision-making processes. As a result of higher education, some study participants viewed these masters prepared nurses as automatically being more involved. One study participant perceived that colleagues with master’s degrees must be engaged in certain change initiatives and projects to improve quality of care and patient safety and/or to train staff nurses about evidence based practice. For example how to formulate a PICO (e.g. population, intervention, control, outcome) or searching for evidence for certain nursing care issues in daily practice.

### Challenges for hospital policy

The respondents most often mentioned lack of information as a key challenge to empowerment. They reported that information was available but that it was fragmented, and that only certain colleagues and committees were aware of it. Staff nurses had to take initiative to gather the necessary information. Participants reported the need for better communication and most staff nurses perceived that this lack of communication was associated with failure of top-level management to promote hospital policies. Otherwise, various initiatives had been taken step by step, using a certain communication plan (e.g. intranet, email).

Staff nurses with a master’s degree found that communication means more than providing information; it also provides feedback about ongoing projects and decision-making processes. Therefore, this is an important issue to be solved by hospital internal governance and policy mechanisms. Participants agreed that experiences of involvement within a large workforce was challenging. Many felt that hospital management teams did a good job, but that there were still opportunities for improvement. One participant noticed changes within the hospital hierarchical approach and structure, reporting that these structures were diminishing “little by little”. The hospital clearly has the aim to go forward. Systematically staff nurses have more ability and power.

*They (e*.*g*. *nursing and hospital management) create a lot of challenges for involvement and yes*, *… the hierarchical structure little by little is vanishing*. *A lot of tasks have been pushed down*, *staff nurses and other healthcare workers have more responsibility*. *I like it very much*, *more responsibilities means that I can decide and do more instead of just acting on what others have decided*. *But too many tasks are pushed down*, *after a while I think … ouch … (Interviewee 5)*.

Although there were favorable perceptions and experiences about the hospital internal governance and policy, some staff nurses felt empowerment as an obligation. The opportunities and support *the productive ward* initiative provided about empowerment and involvement were experienced as being contradictory with the *JCI-accreditation* requirements, and therefore some staff nurses felt empowerment as a top-down approach. The *productive ward* process provided staff nurses with opportunities to decide for themselves *how work would be done on their unit* within the *Lean philosophy* (eliminate waste and create more added value for patients). But the *JCI-accreditation* requirements were perceived as more top down implementation and made staff nurses feel that they could only make decisions when hospital management allowed them to do so.

*We have started with the productive ward program as part of JCI-accreditation and often I feel that we have to follow one direction without the opportunity to deviate (Interviewee 2)*.

Lack of time was also mentioned as a challenge to empowerment. While top-level management expected that nurses should take initiatives and solve problems, the staff perceived the lack of sufficient time to do so. The majority of the participants recognized that not all staff nurses on their unit were pleased with empowerment. Some colleagues were not interested in more challenges and responsibilities and joint decision-making; these colleagues did not see opportunities associated with empowerment and may have experienced empowerment as demanding.

*I think that not all colleagues were pleased and some may have experienced empowerment as additional demands*, *… you have to do more*. *Some colleagues like the classic nurse role*, *… tell me what I have to do (Interviewee 6)*.

## Discussion

Based on semi-structured interviews of 11 staff nurses practicing on medical and surgical units in a university hospital in Belgium that had an initiative in effect to promote nurse empowerment, study results showed that nurses experienced conditions of empowerment. According to participant responses, empowerment was not a usual lexicon and the meaning of empowerment was not well known. However, staff nurses did recognize that nursing and hospital management was initiating several strategies to improve nurse involvement in decision-making processes. Moreover, study findings underpinned Kanters’ sources of empowerment [2]. Respondents reported aspects of formal and informal power through involvement in decision-making processes within their teams highly related to patients and care matters. Although not always clear, there was access to information about ongoing change initiatives to support unit decision-making processes. Certain initiatives such as access to scientific databases and communication strategies that guided change initiatives were present. Nurses reported that opportunities to learn and personal development through several training programs and workshops were highly supported by the hospital. Supportive peer relationships as well as supportive nurse managers’ were experienced.

Staff nurses preferred to be involved in policies related to nursing practice and patient related care, however, they were less willing to be involved in hospital internal governance and policy issues. This finding demonstrated that even though the hospital was in the midst of a transformation process to move from classic hierarchical and departmental based organization to one that was flat and interdisciplinary, that a gap between practice and management levels was still present. However, staff nurses reported that the hierarchical approaches and structures were diminishing “little by little”. Nursing unit manager support as well as nursing team climate were viewed as key factors to develop empowerment and joint decision-making. Good relationships with the unit nurse manager and between peers practicing on the nursing unit were viewed as key to support empowerment. Participants viewed physicians as not being very involved in developing nurse empowerment although staff nurses reported open and supportive relationships with physicians.

Negative aspects of empowerment and more involvement were also reported. Staff nurses mentioned that certain top-down initiatives created confusion and misunderstanding and that they were contradictory to the involvement that was created by *the productive ward program*. Staff nurses reported that this program had a strong focus on their daily practice and patient care within nursing teams, while they felt less engaged in broader hospital initiatives such as the accreditation process. Therefore, some staff nurses felt that empowerment was obligated by the hospital management and thus experienced it as an additional demand. Otherwise, lack of time within daily practice was generally mentioned as a threat, to quality direct patient care. Staff nurses did report desiring to deliver the best possible patient care. However, the impact of empowerment on patient care quality was not clear to participants because several change initiatives taking place in the hospital at the same time and the effect of these initiatives was not yet known.

Our study results are in line with previous studies. A longitudinal study based on surveys with 545 staff nurses from 49 hospital units in Canada [3] identified that individual factors such as psychological empowerment and core self-evaluation as well as contextual factors such as structural empowerment and organizational resources impact nurses’ job satisfaction and unit effectiveness. A Korean study of 340 nurses showed that empowerment mediated the relationship between job characteristics, transformational leadership and work effectiveness [36]. Another Canadian study [37] showed the impact of resonant leadership and individual empowerment on spirit at work (e.g. nurses’ individual experiences that energized their work), job satisfaction and organizational commitment. A Chinese study [38] confirmed that favorable practice environments impact work engagement through psychological empowerment. Another Chinese study [39] confirmed that structural empowerment facilitates the professional practice environment and nurses’ commitment. As reported by our study participants, Davies et al [40] showed the pivotal role of managers in supporting empowering work environments that are conducive to the transfer of knowledge in practice and evidence-based care.

Our study identified the perceived positive and negative aspects of structural empowerment. The positive aspects are in line with the findings from our longitudinal study [22] as well as previous studies that clarify how nurses can be involved through joint decision making and participation in change initiatives on their clinical practice units or hospital-wide and the positive impact on nurse and patient outcomes. However the findings we report in this article also identified negative perceptions and experiences. Top-down decisions and approaches may be confusing difficult for staff nurses to accept.

Our study identified the difficulties one hospital encountered during an ongoing process to transform to a flat and interdisciplinary organizational structure. Our findings provide information gleaned from insights and knowledge from healthcare workers’ feedback to be considered during such transformational processes. Such initiatives present a critical learning process for all stakeholders, executives, nurse managers, and physicians as well as staff nurses.

### Limitations

We recognize that several limitations have to be considered when interpreting and translating these research findings. Firstly, the study was performed in one hospital with an ongoing organizational transformation process. This may limit transferability of the findings. Thus, further research is needed to confirm the identified themes in other settings. Secondly, because of the selection of typical cases (medical/surgical units); we excluded other types of clinical units from our sample. Therefore, we have no information about nurse perceptions about structural empowerment on specialized patient care units such as intensive care and operation theatre. Thirdly, we investigated nurses’ perceptions and experiences about structural empowerment but we cannot make any statements about psychological empowerment. Further research is needed to understand how aspects of structural empowerment affect nurses’ psychological empowerment. Finally, relatively new on-going change initiatives were in operation in this hospital during the time of our study and we were unable to fully understand their impact on nurses, their perceptions and the care they provided.

## Conclusion

This research used a qualitative design that incorporated semi-structured interviews to better understand nurses’ perceptions and experiences about structural empowerment. Both positive and negative aspects of empowerment were identified. Staff nurses recognized the opportunities structural empowerment provided within their daily practice but they were not fully aware of the challenges that may impact quality of care and patient safety. Nursing unit managers and unit climate were seen as crucial to facilitate successful empowerment initiatives while a lack of time and perceived work demands were seen as barriers to nurse empowerment. Our research result confirm the outcomes of various quantitative studies and provides additional and more detailed information about possible negative aspects and potential threats surrounding hospital organizational transformation.

## Supporting Information

S1 File (PDF)
