# Supplementary material for: Staff Nurses’ Perceptions and Experiences about Structural Empowerment: A Qualitative Phenomenological Study
Source: PLoS One. 2016 Apr 1;11(4):e0152654. doi: 10.1371/journal.pone.0152654 (PMC4818078; doi:10.1371/journal.pone.0152654)
Supplement: S1 File — (PDF) [file pone.0152654.s001.pdf]

Written interview analysis and open coding first investigator

Als ik empowerment hoor, dan zou ik denken aan improvement. Dat is een ander woord, dan zou ik denken aan vernieuwing	empowerment -improvement
En met dat improving niet empowerment is, zou ik denken van empowerment dat is eigenlijk de kracht dat ze er insteken om iets te vernieuwen. Dat denk ik, maar meer heb ik daar niet een gedacht van van wat het is	empowerment is de kracht die ze erinsteken om iets te vernieuwen.
Ah ja nee, gewoon , empowerment..improvement allez ja omdat ik dat zo vergelijk eigenlijk	empowerment-improvement een vergelijking
Ja, ik vind wel.. dat het UZA, in onze dienst wel aan die empowerment werkt.	onze dienst wordt wel aan empowerment gewerkt.
We krijgen de kansen en de inspraak voor dingen te zeggen en..ja, zo wel op dienst , vind ik ehm….ja,hoe kan ik een voorbeeld geven?	krijgen de kans en de inspraak op dingen.
Ik zeg maar iets, met nu gaan ze, het JCI project..eh, dat zijn opdrachten die wij moeten vervullen, eh, die van bovenaf komen maar dat zijn ook dinges	JCI poject: opdrachten te vervullen van bovenaf maar waar wij wel inspraak over hebben..
waar we onze ideeën zelf kunnen over loslaten. Ja, dat zou ik zeggen, ja dat is die empowerment	Ideeën kunnen loslaten: empowerment.
Ja, ja, ik vind dat wel…	ervaring met empowerment
Ja, ja, en ik vind de laatste tijd meer, dat dat zo meer ook verlangd wordt dat je daar in….ja…	wordt laatste tijd meer en meer toegepast, wordt ook van je verlangd.
Vindt U dat dit een positieve invloed heeft? Of een negatieve invloed dat empowerment? Legt dat druk op U of …	
Oh, ik vind dat een beetje aanpassen, want we zijn dat ook.Nu is dat zo gelijk ja,	is een beetje aanpassen: 10 jaar geleden was het niet zo.
.och, ik zeg maar iets , een tiental jaar geleden was dat zo niet. Dan was er zo af en toe eens een vernieuwing en dan had je d'er wel wat tijd voor en dan werd dat zachtjes ingeburgerd.	vroeger was het niet zo: af en toe vernieuwing, meer tijd, zachtjes ingeburgerd
..'t zijn meer stappen ..eh 't wordt wel .. en het wordt ook verlangd dat je d'er mee meedoet	nu: meer stappen, en het wordt verlangd

Ja, eigenlijk wel, ja	toch positief
Ja, allez, ja, ik word er graag in betrokken .. eh, maar soms heb ik zo wel ietske van . oh, weeral iets.. dat altijd wel iets , iets erbij is. Maar ik vind dat wel positief, ja, ja..	graag in betrokken maar toch gevoel soms van: weeral iets,,,
Ik denk dat het grootste deel wel komt van de leidinggevenden, van onze hoofdverpleegkundige,,	mogelijkheden tot empowerment komt grootste deel van de leidinggevende: hoofdverpleegkundige
die waarschijnlijk de opdrachten krijgt om dat zowat over te brengen.	hoofdverpleegkundigen opdracht om empowerement over te brengen
Eeh, qua dokters…….ja, neen, daar heb ik er zo geen dingen op dat die daar echt..dat valt niet op dat die daar echt mee bezig zijn..allez, op het punt waarop we nu zitten eigenlijk hé.	artsen zijn daar niet echt mee bezig, valt toch niet op. Toch niet op het punt waar wij nu zitten.
Euhm, ik denk dat dat wel de bedoeling is dat die ook meewerken want anders ga je niet bekomen wat dat je moet bekomen. E	denk dat bedoeling is dat artsen meewerken
en dan eigenlijk dan in gans onze ploeg zijn er eigenlijk een aantal mensen die dat zo wat in hen hebben om dat zowat te stimuleren en en voor u zowat goesting te doen krijgen…..	mogelijkheden tot empowement door mensen in ploeg die dat zowat in hun bebben en stimuleren en goesting doen krijgen.
Ja, leiders die zo wat, ja, je hebt mensen die dat als ze een opdracht krijgen daar zo direct mee weg kunnen.	leiders in team die met opdracht direct weg kunnen.
En je hebt anderen, zoals ik, die dat eerst wat moeten laten bezinken en ik zal wel meedoen, maar ja, ik moet eerst goed weten hoe dat het in één zit.	anderen moeten opdracht eerts laten bezinken. Wel meedoen maar eerst goed weten hoe het ineen zit.
Ja, ik vind dat wel.	collega's stimuleren.
Ik vind dat je elkander kunt stimuleren	
Want als iedereen zegt:pffff ik doe iets niet of hé , natuurlijk iets nieuw is iets dat je moet aanleren en waar je je moet aan aanpassen,	iets nieuws is iets wat je moet aanleren, aanpassen.
maar als iedereen een kijk heeft van : kom aan we gaan dat doen, jaa..	iedereen een kijk heeft: kom aan we gaan dat doen

Goh, uiteindelijk stellen ze hun..ja,hoe moet ik het zeggen…hun projecten eigenlijk voor in ne keer een voorstelling, gelijk bijvoorbeeld 'the eight hours' worden er veel dingen gezegd: van dat komt er, hé, en dat gaat terug komen en dat gaat terugkomen, dus dan kan je het al ne keer laten bezinken.	beleid stelt projecten een keer voor bv in de eight hours .
Meestal diene eerste dingen is van : ach wat gaan ze nu weer doen,hé.	Wat gaan ze nu weer doen?
Euh, maar dan eigenlijk wordt dat stap voor stap aangebracht en ja, er zijn ook wel mensen voor vrijgesteld die dat dan eens op dienst komen voorstellen, ja en zo kom je d'er in.	stap voor stap aangebracht
En ze houden U eigenlijk via internet ook op de hoogte van de verschillende punten dus ja…	beleid houdt je op de hoogte via internet van de verschillende punten.
Goh,gebruik? de middelen zijn er maar gebruik van maken….? Dat is dikwijls ne keer tussen door ,hé, gelijk , ja, ik doe avondshiften en nachtdienst en met mijn nachtdiensten heb ik soms ne keer den tijd voor zo ne keer open te klikken en voor ne keer iets te lezen of te doen. Sommige mensen proberen dat ook eens tussen de uren te doen, want ja, na uw uren ga je niet meer op de computer voor 't werk zitten hé. Dat is enkel op 't werk dat je dat kunt, ja	middelen zijn er maar dikwijls tussendoor gebruik van gemaakt. Vb avond- of nachtshift.
.ik heb wel de indruk dat er wordt gelezen wat dat er moet gelezen worden	er wordt gelezen wat er gelezen moet worden.
Ja, ja, ja,	beleid doet moeite om in te lichten
Hoe worden dingen beslist? Komt dat van hogerhand? Of kunnen jullie zelf op de dienst zaken beslissen waarvan je zegt: dat zou beter zijn voor de kwaliteit van zorg voor de patiënt?	
Goh, al er iets beslist wordt of euh, hoe moet ik dat zeggen… euh, als er iets nieuws is of iets waarvan we ons moeten gaan aanpassen, daar krijgen we dikwijls een mail van hé,	beslissingen worden dikwijls via mail gecommuniceerd.

en als we daar commentaar op hebben of bedenkingen ja, dan zeggen wij dat tegen onze hoofdverpleegkundige .Zijn dat echt dingen die niet, euh waar dat totaal niet mee….ik kan zo niet direct een voorbeeld van iets geven. Als het nu iets is waarvan je niet mee akkoord gaat dan ga je naar je hoofdverpleegkundige en die kan dan stappen, of op een vergadering eens zeggen: ze gaan er niet mee akkoord.	commentaar en bedenkingen aan hoofdverpleegkundige melden.
Ja, euh……ja dus euh bijvoorjbeeld…. Ik sta op XXX en wij hebben verschillende specialiteiten . In , onder handde van pijn euh..catheters en ja, het zijn zo allemaal post operatieve patiënten euh, heeft anesthesie al wel ne keer info gegeven omdat we zeiden van: hoe zit dat met die catheter, is dat normaal dat die mensen zo lang geen gevoel hebben ? Hé, dan is daar zo een aparte voordracht van gegeven. Euh, XXX geeft ook wel eens informatie.	info gegeven door anesthesie ivm catheters/pijn.
Informatie Ja, ja, daar wordt soms ook ne keer van ons uit ook gevraagd, zenne. Hoe zit dat nu met dat ….	vanuit verpleegkunde de vraag om info: wordt op ingegaan.
Ja, ja,toch wel. Als je erom vraagt, dan gaan ze dat wel doen.	invloed als je erom vaagt gaan ze dat doen.
Naar de dokters toe eigenlijk , als je een vraag hebt kunt ge altijd terecht, maar je moet ze stellen hé.	naar artsen toe kan je vragen stellen maar je moet ze wel stellen.
Dat heb ik nu persoonlijk nog niet meegemaakt. Maar ik denk dat als er iets is en je kan het bewijzen hé, dat je zegt van ja ik heb dat gelezen of wetenschappelijk ding gelezen. Ik denk wel dat er personen zijn waardat je bij langs kunt gaan. Ik, bijvoorbeeld, als ik nu zeg de wondzorg…ik ga daar niet mee akkoord met dat protocol …voor ja,.. bijvoorbeeld het ‘ ros’ (?) systeem bijvoorbeeld, ja en ik kan dat verantwoorden dat ik gelezen heb dat dat beter zou kunnen, of anders zou kunnen, dan zou ik contact opnemen met de wondverpleegkundige .	indien je iets (wetenschappelijk) kan bewijzen, kan je wel bij bepaalde personen terecht vb wondverpleegkundige.
Ja, maar ik denk ook dat dat van u eigen moet afhangen,euh, van u eigen moet komen, van wil ik daar de moeite voor doen op daarop in te gaan of blijf ik gewoon het protocol volgen, gelijk dat mij opgelegd wordt.	invloed op beslissingsprocessen kan maar hang van je eigen af. Wil ik daar de moeite voor doen?
Ik denk echt dat als je met iets niet akkoord zijt, dat je het wel tegen iemand kunt zeggen en dat er wel geluisterd wordt. Of dat het veranderd wordt, ja, dat hangt ervan af hoe goed euh, dat je uw dingen euh, kunt staven.	indien niet akkoord kan je het iemand zeggen en wordt er geluisterd. Of het veranderd hangt af van het staven van je argument.

Ja, uiteindelijk ik vind zijn die standing orders, als dat uitgaat van de artsen en dat is wat samen met verpleegkundigen euh, opgesteld, dan vind ik dat wij als andere verpleegkundigen ons daaraan, ja daarvoor zijn het ook standing orders…	geen invloed willen hebben op standing orders. Zaken worden opgesteld om te volgen.
en dat geeft ook voor een stuk een gerustheid dat je weet dat is een standing order, ik hou mij daaraan en ja allez, ja…ik gebruik dat.	gerustheid door standing orders
Neen	geen interesse in werkgroep
Awel, ik vind dat we dat nu eigenlijk hebben met euh.. de verschillende omvormingen van onze lokalen die we moeten doen. Daar hebben we nu ons volle inzeggenschap, allez , ons volle, toch een groot stuk ons zeggenschap in en kunnen we zeggen van ja, we willen het zo en we willen het zo En dat vind ik al allez, ja, de werkomgeving die we wat kunnen aan passen. Dat vind ik echt euh…..	eigen beslsissingen mogelijk in directe werkomgeving: omvorming lokalen, groot stuk zeggenscahp.
Ja, daar wil ik mee over beslissen omdat ik daar ook dagelijks in werk en euh,	meebeslissen omdat ik daar dagelijks in werk.
dat ik daar ook het nut van zie en de positieve dingen ervaar .	meebeslissen omdat ik er het nu van inzie en de positieve dingen ervan ervaar.
Als we het kunnen schikken naar waar we het het gemakkelijkste vinden.	meebeslissen naar wat we als gemakkelijkste ervaren.
Ja, naar patiëntveiligheid bijvoorbeeld. Ja, de beslissingen die genomen worden zijn ook wel goed hé. Euh..ja…goh…patiëntveiligheid ja? De verschillende dingen waardat we rekening mee houden hé.	goede beslissingen aangaande patiëntveiligheid.
Zoals de armbandjes…ja, die worden nu omgedaan op de opnamendienst. D'er, er is ons gezegd van ja, boven mondeling te controleren zowel de stickers als de armbandjes . Dat wordt gedaan	procedure patiëntveiligheid ivm identificatie wordt opgevolgd.:
, en ik heb de indruk dat dat een veilig gevoel geeft bij de patiënten en .. allez, voor dat te doen vind ik dat ook zo iets van , ja, je checkt dat en je geeft ze toch zo iets van vertrouwen , ja ……patiënten ja, patiënt veiligheid.	veilig gevoel bij patiënten. Je geeft patiënt vertrouwen.
Ja maar, als je je werk beter kan organiseren en vlotter kunt doen, dan vind ik dat ook een positief punt voor de kwaliteit van zorg naar de patiënt toe.	werk beter georganiseer en vlotter draagt positief bij tot kwaliteit van zorg naar patiënt toe.

Als je vlotter werkt en je vindt sneller uw gerief bijvoorbeeld, of .. je weet welke procedure of standing order dat je je kan houden dat geeft allemaal een zekerheid en ook wel vertrouwen bij uw patiënt, dat hij ziet dat je weet met wat je bezig zijt	Vlotter werken, weten welke procedure, of standing order geeft een zekerheid. Vlotter werken , weten welke procedure geeft meer vertrouwen bij uw patiënt en pt ziet dat je weet waar je mee bezig bent.
Dat je niet iets, als ze je een vraag stellen, dat je niet iets moet gaan vragen maar dat je er gewoon kunt naartoe gaan en ja, zeggen : kijk, ik moet dat doen, dat, dat dat …Dat geeft toch vertrouwen? Ja ik denk dat wel …	door te weten wat je moet doen, en niet opnieuw moet gaan vragen, geeft vertrouwen aan patiënt
Ja, toch wel….	mee beslissen draagt bij tot kwaliteit?
Nee, nee, nee, ik vind dat van hogerhand moeten ze, en kunnen ze, allez ja, dat is hun recht want ze kunnen van hogerhand dingen opleggen hé.	va hogerhand moeten, kunnen, is het hun recht om dingen op te leggen.
Euh , Als verpleegkundige is 't eigenlijk uw plicht van het te volgen . Ja, als je zegt ik werk in die organisatie, dan moet je de dingen volgen van hogerhand	als verpleegkundige is plicht om dingen te volgen van hogerhand
maar het is wel uw recht om te zeggen van ja, maar ik ga daar niet volledig mee akkoord en ik ga daar wel volledig mee akkoord maar dat je inspraak hebt, ja…	wel recht om te zeggen dat je er niet mee akkoord gaat, inspraak krijgen.
Ja, je voelt u meetellen hé	belangrijk dat je gevoel hebt dat je meetelt,
Ja, voor mij is dat toch belangrijk dat je het gevoel hebt van ja, er wordt geluisterd naar mij.	belang rijk dat je het gevoel hebt dat er geluisterd wordt naar mij.
Goh, meer inspraak ? Nee.	niet meer inspraak dan 10 jaar geleden.
Nee, dat zou ik niet durven zeggen, nee want toen ik hier juist begon vond ik dat er ook geluisterd werd.	er wordt niet meer geluisterd dan 10 jaar geleden. Toen werd er ook geluisterd.
Het is niet dat ze extra moeite hebben gedaan om je meer te betrekken?	
Nee. Nu draait het meer al om communicatie . Nu wordt er over alles gebabbeld	beleid doet geen extra moeite om ons meer te betrekken. Nu draait het meer om communicatie
eeuh, er wordt van alles mails doorgestuurd ..allez, ja, je wordt meer op de hoogte gehouden.	.Je wordt meer op de hoogte gehouden

Ja, en ik denk dat dat ook gewoon een evolutie is van de tijd.	beter geïnformeerd evolutie van deze tijd.
Ja, zo kun je dat zien.	empowerment zorgt voor meer betrokkenheid in de beslissingsprocessen
Ja toch wel, je bent een stuk verplicht, tussen aanhalingstekens, om te volgen hé. Van mee te werken aan de projecten hé. Ja toch wel.	stuk 'verplicht' om mee te werken aan projecten.
Tja, sterker in mijn schoenen? Ja, misschien wel een stuk omdat je doet kennis op en je werkt aan zaken, ja , misschien wel, ja	misschien stukje beter in de schoenen staan omdat je kennis opdoet en meewerkt aan zaken,
Ja, ik denk toch wel.	voelt zich sterker om job uit te voeren
Ja, ik zo wel durven zeggen van ja.	krijgt een stem in beslissingsprocessen
…..Ik vind dat er voldoende wordt geëvalueerd na een project of zo om te vragen wat je er van vond.	ziekenhuisbeleid evalueert voldoende na een project.
Dus nog meer? Allez Ja, het mag altijd meer, als ze persoonlijk komen vragen, ja, het mag meer maar het hoeft niet meer	mag altijd meer vb persoonlijk. Maar het hoeft niet.
In het begin is dat … belastend is wel een groot woord hé.	vernieuwingen beslastend voor werk is een groot woord. In begin wel
Ge zijt er mee bezig en nieuwe dingen moet je altijd aan aanpassen en leren of ja, dat is zo…	nieuwe dingen vragen altijd aanpassen en leren.
euhm ik heb wel de indruk dat mensen die juist van het school komen en zo, er meer euh, nog meer voor openstaan.	schoolverlaters staan meer open voor nieuwe dingen
Ik vind dat ik zo in de tussendingen zit , niet meer bij de oudere collega’s, niet meer bij de jongere, zo er tussen. Ik vind dat ik er redelijk mee weg kan	tussen jonge en oudere collega's: redelijk goed mee weg met vernieuwingen.
maar het valt mij ook op dat er wel oudere collega’s er wel soms meer moeite mee hebben om nieuwe dingen aan te nemen en..mee te werken.	oudere collega's meer moeite om nieuwe dingen aan te nemen en mee te werken.

Misschien wel, omdat het telkens iets nieuw is en iets dat je moet aanleren, ja , allez, nu denk ik aan het systeem van ja, gewoon dat alles via de computer komt. Dat is voor sommige generaties iets nieuw ook hé. Wij zijn volop bezig met GSM's en dingen, dus wij tikken en wij doen maar, wij kennen dat. Ja, maar dat is soms wat moeilijker , ja	misschien te snel na elkaar nieuwe zaken aan te leren voor oudere collega's.
Nee, dat denk ik niet, … ik denk dat ze dat wat meer als druk zien.	denkt dat oudere collega's wat meer druk zien.
Ik zie dat niet echt als druk, ik zie dat als ja, OK, iets dat moet vernieuwen en iets dat we allemaal moeten doen, en OK, en ik zit er mee in, maar ik zie dat niet echt als druk.	ziet het niet echt als druk dat er iets vernieuwd maar als iets wat we allemaal moeten doen en zit er mee in.
Ja, ik denk dat wel, want er wordt er bewuster mee omgegaan. Er wordt er eens bij stilgestaan.	vernieuwingen dragen bij tot kwaliteitsvolle en veilige zorg want er wordt bewuster mee omgegaan.
En er wordt ne keer, ja, alles wordt een keer bekeken: wat kunnen we doen voor die veiligheid naar de patiënt toe. Wat zou er interessanter zijn? Naar protocols toe, naar veiligheid toe, ja	er wordt een keer gekeken : wat kunnen we doen voor de veiligheid van de pt. Wat zou er interessanter zijn?
Ja, ik moet wel zeggen dat ik vind het geen last. Ik vind het ook niet erg van ermee bezig te zijn maar ..soms heb ik zo iets van…euhm de patiëntenzorg, de echte zorg, ja..daar wil ik eigenlijk meer tijd insteken.	ondervind geen last van empowerment bij werktevredenheid. Soms wel wens voor meer tijd voor patiëntenzorg.
In de zorg. En euh, OK als er dan gezegd wordt: je moet diene mail nog lezen of dat rapport moet nog ingetikt worden of, of,… ja dan denk ik soms van och, die extra minuten zo gewoon een babbel met uw patiënt, dat vind ik ook belangrijk.	extra tijd voor babbel met patiënt is soms belangrijk ipv tijd voor informatie en vernieuwingen.
Ik zeg maar iets, de protocols van de dienst. Daar wordt toch uw mening over gevraagd, van dat een keer te lezen, en te zeggen moeten er aanpassingen gebeuren of niet. Ja…	betrokken worden in bv protocols draagt bij tot werktevredenheid: er wordt naar uw mening gevraagd
Ik vind dat elke inspraak bijdraagt tot uw werktevredenheid omdat ja, ze vragen u van akkoord, niet akkoord, ja	elke inspraak draagt bij tot werktevredenheid.
Wel, ik denk dat het moment dat je de druk begint te voelen en dat u daardoor, ja …verplicht voelt ja of niet meer goed, allez, niet meer goed voelt en er niet mee akkoord kunt gaan	intentie tot verlaten job: als je druk begint te voelen, niet meer goed voelt en niet meer akkoord kunt gaan.

en het gevoel hebt: als ik dat zeg, wordt er niet meer naar mij geluisterd. Ik denkt dat als je op dat punt komt, ja….	intentie tot verlaten job: ik zeg iets en er wordt niet meer naar mij geluisterd.
Dan denk ik wel dat dat marcheerd, ja	luisteren en nodige ondersteuning marcheert.
Ja, ja maar ik denk ook even goed negatief als het gewoon van hogerhand zo en zo en niet anders zonder dat er niet een keer aan u gevraagd wordt van zeg: wat vindt u hiervan of, ja.. dan denk ik dat dat negatief zou kunnen werken.En, en dingen afstompend, allez, ja, afstompend, allez, demotiverend zou kunnen werken.	geen empowerment of betrokkenheid van hogerhand werkt negatief: zou afstompend, demotiverend kunnen werken.
Ja, ik denk dat in het maken van die procedures dat dat een zekerheid geeft van dat je weet van ik volg hetgene wat moet gevolgd worden, ik doe het zoals het moet..	gevoel van meer capaciteiten door maken van procedures want geeft meer zekerheid over wat je doet.
Ja, maar ik had dat daarvoor ook al, Ik heb zeker niet meer vertrouwen.	niet meer vertrouwen dan vroeger.
Nee, nee.. ik vind het wel positief dat er zo eens naar gevraagd wordt.ja, zodat je je het zelf eens kan zeggen.	positief dat verpleegkundigen bevraagt worden en dat je het zelf eens kan zeggen.
Wat ik eventueel toch even zou willen zeggen, ik ben van opleiding A2 verpleegkundige, hé, dus zoveel jaar geleden had je A1 en A2. Nu heb je mensen die gaan voor een bachelor. Ik heb zoveel jaar geleden geprobeerd die bachelor tedoen, toen heb ik gezien dat ze, ook over die management, dat ze daar veel andere dingen inkwamen dan toen mijn A2 opleiding. En dat er toen veel mensen van de A opleiding zeiden dat diene bachelor is ook al iets totaal anders hé. En nu doen de mensen de master, dat is nog stap hoger , die zijn daar zo eigenlijk allemaal veel vlugger mee weg. Allez, ja, die hebben zo allemaal al meer die info gehad	verschil in A1 en A2 vroeger en nu. Bachelor nu management, veel andere dingen dan vroeger A2 of A1. Nu master opleiding. Deze mensen zijn daar allemaal veel vlugger mee weg. Meer info gekregen.
Zo over die vernieuwingen , over hoe het moet draaien en het reilen en het zeilen	bachelors en masters meer info over reilen en zielen van de vernieuwingen.
Ja, maar die indruk heb ik wel , dat wordt gedaan hé. Ja , die dagen, bijvoorbeeld die 'eight hours' en dan, allez omdat ik daar een stukje van geproefd heb van die bachelor hoor ik daar veel dingen van terugkomen.	wel indruk dat er iets aan gedaan wordt om andere verpleegkundigen ook te informeren over beleid: eight hours.

En kun je iets hebben van , ja, als je die opleiding gehad hebt, heb je een stapje voor en dan ga je daar nog opener voor staan voor al die vernieuwingen omdat je dat al een stuk in je opleiding gehad hebt. En dat heb ik zo niet gehad in mijn opleiding	wie bachelor, master opleiding heeft gehad, heeft stukje voorsprong en staan opener voor al die vernieuwingen omdat er al een stuk in opleiding was.
Ik denk dat wel	gemis door oudere werkernemers van voorkennis die afgestudeerd bachelors al hebben door opleiding.
Ik denk dat wel, ja, allez, nu wordt dat er al voor een stuk ingebakken en, en is dat een onderdeel van de opleiding verpleegkunde.	denkt dat het (vernieuwingen) hierdoor (gemis aan voorkennis tov bachelors) moeilijker is voor oudere werknemers
Ja, ja, zij hebben al meer achtergrond; En het is wat ik daar juist zei van diene zorg . Ik vond in ons opleiding stond dat heel centraal . Het contact en de zorg en de…. ja	Bachelors voordeel omdat ze al achtergrond hebben. Vroeger opleiding stond zorg centraal.
Achterwege zou ik nog niet durven zeggen want ik zou zelf durven zeggen dat, dat de kwaliteit ten goede komt omdat ook kennis.. je gaat kritischer kijken naar dingen, dat wel. Maar de tijd misschien… denk ik	door andere opleiding geen mindere kwaliteit van zorg , integendeel komt kwaliteit ten goede. Kritischer kijken
Ja, de gewone zorgtijd. Niet dat je niet met die patiënt bezig zijt, want ge zijt bezig ook al sta je voor die computer, het is voor de patiënt dat je bezig zijt. Maar het menselijke , zo ja, ..het zorgen	de gewone zorgtijd komt, het menselijke komt in gedrang.
Nee, niet door te laten doen . Nee gewoon door de dingen die erbij komen euh, ja dingen waar dat je mee moet bezig zijn , ja, ja, … dat wou ik nog eventjes zeggen	door dingen die erbij komen gemis aan relatie met patiënt tot uiting.

Written interview analysis open coding and themes second investigator

Aan improvement, aan vernieuwing	Improvement, vernieuwing.	improvement	definitie empowerment
		vernieuwing	definitie empowerment
De kracht dat ze erin steken om iets te vernieuwen.	De kracht om iets te vernieuwen.	kracht	definitie empowerment
Wel aan empowerment werkt. De kansen en de inspraak om dingen te zeggen. JCI-project dat zijn opdrachten die wij moeten vervullen, die van bovenaf komen, maar dat zijn ook dingen waar wij inspraak over hebben. Onze ideeen zelf kunnen loslaten.	Kansen en inspraak om dingen te zeggen. In JCI-project hebben wij inspraak, kunnen onze ideeen loslaten.	kansen	definitie empowerment
		inspraak	definitie empowerment
		ideeën loslaten	definitie empowerment
		JCI project : inspraak	projecten
Ja, ik vind dat wel ervaring heb.		wel ervaring mee	gevoel van ervaring met empowerment
Een beetje aanpassen. Tienjaar geleden wat dat niet, dan was er af en toe een vernieuwing, dan had je er wel tijd voor en werd dat zachtjes ingeburgerd. Nu is dat gelijk meer stappen, wordt verlangd dat je mee doet.	Tienjaar geleden werden vernieuwingen zachtjes ingeburgerd, nu is dat gelijk meer stappen en wordt verlangd dat je mee doet.	vernieuwingen zachtjes ingeburgerd	vroeger vernieuwingen
		meer stappen	hedendaagse verniewingen
		verlangd dat je meedoet	hedendaagse verniewingen
		verlangd dat je meedoet	wil van beleid
Ja, vind dat positief.		positief	positief tov empowerment

Ik word er graag in betrokken, maar ik heb wel zoiets van weeral iets, iets erbij. Ik vind dat wel positief ja.	Wel graag in betrokken, maar wel zoiets van weeral iets erbij.	graag betrokken	positief tov empowerment
		maar wel zoiets van weeral iets erbij	negatief tov empowerment
Grootste deel van de leidinggevenden, onze hoofdverpleegkundigen. Qua dokters, valt niet op dat die daarmee bezig zijn. Ik denk dat dat wel de bedoeling is dat die ook meerwerken, want anders ga je niet bekomen wat je moet bekomen. In onze ploeg een aantal mensen die dat zo wat stimuleren.	Grootste deel komt van onze hoofdverpleegkundigen. Valt niet op dat de dokters daarmee bezig zijn. Ik denk wel de bedoeling dat die meerwerken, anders ga je niet bekomen wat je moet bekomen.	grootste deel komt van hoofdvpk	kansen tot empowerment door hoofdverpleegkundige
		zijn daar niet mee bezig	kansen tot empowerment door artsen
		wel bedoeling dat artsen meewerken anders ga je niet bekomen wat je wil bekomen	vereisten tot empowerment
Leiders die daar direct mee weg kunnnen En anderen die het eerst wat moeten laten bezinken en dan wel meedoen. Eerst goed weten hoe dat het in een zit.	Leiders die daar direct mee wegkunnen, anderen moeten het eerst laten bezinken en dan meedoen. Eerst goed weten hoe dat het in een zit.	leiders: direct mee weg.	capaciteiten team tot empowerment
		anderen: laten bezinken	capaciteiten team tot empowerment
		anderen: eerst woeten hoe het zit	capaciteiten team tot empowerment
Ik vind dat je elkander kunt stimuleren.	Elkander stimuleren.	elkaar stimuleren	capaciteiten team tot empowerment

Iets nieuws, moet je aanleren, aanpasssen.	Iets nieuws moet je aanleren, aanpassen.	aanleren	capaciteiten team tot empowerment
		aanpassen	capaciteiten team tot empowerment
		aanleren en aanpassen	vernieuwingen
Ze stellen hun projecten voor, dan kan je het al een keer laten bezinken. Eigenlijk wordt dat stap voor stap aangebracht. Ook mensen voor vrijgesteld die dat op dienst komen voorstellen, dan kom je er in. Houden ons op de hoogte via internet.	Projecten worden stap voor stap aangebracht. Ook mensen voor vrijgesteld die dat op dienst komen voorstellen. Op de hoogte via internet.	projecten stap voor stap aangebracht	communicatie beleid
		op dienst voorgesteld	communicatie beleid
		via internet	communicatie beleid
De middelen zijn er maar er gebruik van maken? Dikwijls tussendoor. Sommigen proberen tussen de uren te doen, want na je uren ga je niet meer op de computer voor t werk zitten. Dat is enkel op het werk. Wel de indruk dat er wordt gelezen, wat dat er moet gelezen worden.	Er wordt niet altijd gebruik gemaakt van de middelen, dikwijls tussendoor. Proberen tussen de uren te doen, na je uren doet men dat niet meer. Wel de indruk dat wordt gelezen, wat gelezen moet worden.	niet altijd gebruik gemaakt van middelen	gebruik van de middelen
		tussen de uren door	gebruik van de middelen
		indruk dat info gelezen wordt	verwerven info
Ja, beleid doet moeite om in te lichten en mogelijkheden te geven.	Beleid doet moeite om in te lichten.	beleid licht in	communicatie beleid
		doet moeite	inspanningen beleid

Als er iets nieuws is of waarvan we ons moeten gaan aanpassen, dan krijgen we daar een mail van. Commentaar of bedenkingen zeggen we tegen onze hoofdverpleegkundige. Als je echt niet akkoord gaat, dan ga je naar hoofdverpleegkundigen en die kan dan op een vergadering zeggen dat we niet akkoord gaan.	Nieuws en aanpassingen krijgen we via de mail. Commentaar, bedenkingen of niet akkoord worden tegen de hoofdverpleegkundige gezegd.	aanpassingen en niews: mail	communicatie beleid
		aanspreekpunt hoofdverpl	aanspreekpunten
Anesthesie heeft wel een keer info gegeven. Bij vragen een aparte voordracht van gegeven.	Bij vragen wordt er een aparte voordracht gegeven.	voordrachten	kansen tot empowerment door de artsen
Als je erom vraagt, dan gaan ze dat wel doen.	Als je erom vraagt, dan gaat men dat wel doen.	wel erom vragen bij artsen	kansen tot empowerment door de artsen
Naar dokters toe. Als je een vraag hebt kun je altijd terecht, maar je moet ze stellen.		vragen zelf stellen	kansen tot empowerement door de artsen
Persoonlijk nog niet meegemaakt. Ik denk dat als er iets is en je kunt het bewijzen, ik heb dat wetenschappelijk gelezen, dat er wel personen zijn waar dat je langs kunt gaan.	Als er iets is en je kunt het wetenschappelijk bewijzen, dat er wel personen zijn waar je langs kunt gaan.	je kan bij iemand langs gaan ivm EBP	aanspreekpunten
		gevoel van mogelijkheid tot inspraak	inspraak

Van u eigen moet afhangen, van u eigen moet komen. Als je met iets niet akkoord zijt, wel tegen iemand kunt zeggen en dat er wel geluisterd wordt. Of dat het veranderd, dat hangt af van hoe goed je het kunt staven.	Van u eigen afhangen, van u eigen moet komen. Als je niet akkoord bent, dan kun je die tegen iemand zeggen. Of het veranderd hangt af van hoe goed je het kunt staven.	gevoel van mogelijkheid tot inspraak	inspraak
		inspraak hangt van je eigen af.	eigen inbreng
Standing orders, als dat uitgaat van arsten en dat is wat samen met verpleegkundigen opgesteld, dan moeten wij als verpleegkundigen ons daaraan houden. Dat geeft een gerustheid.	Standing orders geven gerustheid.	standings orders: gerustheid	standardisatie : gerustheid
Niet mee beslissen over standing orders.	Niet mee beslissen in standing orders.	geen beslissingen ziekenhuisbreed	niveau van beslissingen
Niet in een team willen zitten om zaken te beslissen die artsen betreffen	Niet over zaken van de artsen beslissen.	geen beslissinen met artsen	niveau van beslissingen
Volle inzeggenschap over de omvormingen van de lokalen. We kunnen zeggen van we willen het zo en we willen het zo.		beslissingen op eigen werkvloer	niveau van beslissingen
Mee beslissen over werkomgeving, omdat ik daar ook in het dagelijks werk het nut van zie, die positieve dingen ervaar.	Van het meebelissen over de werkomgeving, zie je in het dagelijks werk het nut en de positieve dingen.	beslissingen op eigen werkvloer	niveau van beslissingen
		positief gevoel over beslissingen op eigen werkvloer	niveau van beslissingen

Mee beslissen over patientveiligheid.		patiëntveiligheid	niveau van beslissingen
Vb patientveiligheid zijn de armbandjes. Dat wordt gedaan, indruk dat dat een veilig gevoel geeft bij de patienten. Je checkt, geeft ze iets van vertrouwen.	Mee beslissen over patientveiligheid geeft de patienten een veilig gevoel en geeft vertrouwen.	mee beslissen over patiëntveiligheid	patiëntveiligheid
		vertrouwen geven	patiëntgericht empowerment
		veilig gevoel	patiëntgericht empowerment
Als je je werk beter kan organiseren en vlotter kunt doen, dat is een positief punt voor de kwaliteit van zorg naar de patient toe. Vlotter werken, sneller gerief vinden, weten aan welke procedure of standing order je kan houden, geeft dat meer zekerheid en vertrouwen bij de patient. Weet waar je mee bezig bent, dat geeft vertrouwen.	Beter, vlotter, sneller werken is positief voor de kwaliteit van zorg. Procedures of standing order geven zekerheid en vertrouwen.	efficienter werken	kwaliteit van zorg
		procedures /standing orders	standardisatie: meer vertrouwen
		procedures /standing orders	standardisatie: zekerheid
Empowerment draagt bij tot kwaliteit van zorg voor de patient.	Empowerment draagt bij tot kwaliteit van zorg.	empowerment draagt bij	kwaliteit van zorg
Van hogerhand hebben ze het recht om dingen op te leggen. Als verpleegkundige is het je plicht van het te volgen. Het is wel je recht om te zeggen dat je daar niet volledig mee akkoord ben.	Het is je recht om te zeggen dat je daar niet volledig mee akkoord ben.	niet akkoord gaan is recht	inspraak
	hogerhand recht om zaken op te leggen	zaken opleggen	top down

	plicht deze zaken te volgen	plicht	plichten vpk
Door inspraak voel je je meetellen.	Door inspraak voel je je meetellen.	inspraak = meetellen	gevoel van belangrijkheid
Belangrijk dat je het gevoel hebt dat er naar je geluisterd wordt.	Belangrijk dat je het gevoel hetbt dat er geluisterd wordt.	gehoord worden is essentieel	gevoel van belangrijkheid
In 10 jaar niet meer inspraak	niet meer inspraak	inspraak	vroeger
Er werd toen ook al geluisterd.	ook geluisterd	luisterend oor	vroeger
Nu draait het meer om communicatie, je wordt meer op de hoogte gehouden.	nu meer communicatie, meer op de hoogte	meer op de hoogte	communicatie beleid
Meer informatie, betrekken bij beslissingen, ondersteuning geven daarin is een vorm van empowerment.	Meer informatie, betrekken bij beslissingen, ondersteuning geven zijn een vorm van empowerment.	meer informatie	vormen van empowerment
		meer informatie	communicatie beleid
		betrokken bij beslissingen	vormen van empowerment
		ondersteuning geven	vormen van empowerment
Verplicht mee te werken aan de projecten.	verplicht meewerken aan projecten	verplicht meewerken	top down
Misschien sterker in mijn schoenen doordat je kennis opdoet en werkt aan zaken.	Weerbaar doordat je kennis opdoet en werkt aan zaken.	sterker door kennis	effect van empowerment
		sterker door aan zaken te werken	effect van empowerment
Ja, ik voel mij sterker om mijn job uit te voeren, doordat ik gesteund wordt.	Sterker in job, doordat er steun is.	sterker door steun	effect van empowerement
Ja, ik vind dat ik een stem heb in beslissingsprocessen.		stem in beslissingsprocessen	effect van empowerement

Het mag altijd meer, persoonlijk komen vragen. Er wordt al voldoende geevalueerd.	Er wordt voldoende geevalueerd, wel meer persoonlijk komen vragen.	persoonlijk vragen	kansen voor beleid
In het begin is het belastend. Nieuwe dingen moet je altijd aanpassen en leren. Van school komen staan er meer voor open. Oudere collega's meer moeite mee hebben om nieuwe dingen aan te nemen, mee te werken.	In het begin belastend, nieuwe dingen moet je aanpasen en leren. Collega's die van school komen staan er meer voor open en oudere collega's hebben meer moeite om nieuwe dingen aan te nemen, mee te werken.	begin belastend	werklast
		pas van school: meer open	aanpassingsvermogen collega's vernieuwingen
		oudere collega's: meer moeite	aanpassingsvermogen collega's vernieuwingen
Het is telkens iets nieuws, iets dat je moet aanleren. Het systeem van nu is dat alles via de computer komt, voor sommige generaties is de computer ook iets nieuws.	Het is telkens iets nieuws dat je moet aanleren. Nu is alles via de computer en dat is voor sommige generaties ook iets nieuws.	steeds nieuws aan te leren	aanpassingsvermogen collega's vernieuwingen
		sommige generaties veel nieuws	aanpassingsvermogen collega's vernieuwingen
Iets dat moet vernieuwen, iets dat we allemaal moeten doen, ik zie dat niet echt als druk. Sommigen zien dat wat meer als druk.	Sommigen zien iets nieuws als een druk. Ik zie dat niet als druk.	geen druk, sommige wel	aanpassingsvermogen collega's vernieuwingen
Er wordt bewuster mee omgegaan, bij stilgestaan, alles wordt een keer bekeken, naar protocols toe, naar veiligheid toe.	Naar protocols en naar veiligheid toe wordt er bewuster mee omgegaan, bij stilgestaan, herbekeken.	bewuster omgaan met zaken	effect van empowerment

		meer gekeken naar veiligheid	effect van empowerment
		stilstaan, herbekijken	effect van empowerment
Vind het geen last,niet er erg om ermee bezig te zijn. Ik wil eigenlijk meer tijd steken in de patientenzorg, de echte zorg. Die extra minuten een babbel met de patienten, vind ik ook belangrijk.	Geen last of erg om ermee bezig te zijn. Eigenlijk meer tijd steken in patientenzorg, de echte zorg. Een babbel met de patient is belangrijk.	geen last	werklast
		babbel is belangrijk	tijd voor patiënt
		tijd voor echte zorg	tijd voor patiënt
Elke inspraak draagt bij tot werktevredenheid, ze vragen akkoord, of niet akkoord.	Elke inspraak draagt bij tot werktevredenheid.	inspraak draagt bij	werktevredenheid
Het moment dat je druk begint te voelen, verplicht voelt, niet meer goed voelt, niet meer akkoord kunt gaan, het gevoel dat er niet meer naar je wordt geluisterd, die je op dat punt komt van het werk te verlaten.	Het moment dat je druk begint te voelen, verplicht voelt, niet meer goed voelt, niet meer akkoord kunt gaan, het gevoel dat er niet meer naar je wordt geluisterd, die je op dat punt komt van het werk te verlaten.	druk voelen	intentie tot verlaten van werk
		niet goed voelen	intentie tot verlaten van werk
		niet akkoord kunnen gaan	intentie tot verlaten van werk
		gevoel van niet gehoord te worden	intentie tot verlaten van werk

Empowerment kan zowel positief als negatief werken. Als ze van hogerhand iets doen zonder dat het aan u gevraagd wordt, dan zou dat negatief en demotiverend kunnen werken.	Empowerment kan positief als negatief werken. Als ze van hogerhand iets doen zonder dat het aan u gevraagd wordt, dan zou dat negatief en demotiverend kunnen werken.	top down opleggen	negatief effect empowerment
Het maken van procedures geeft zekerheid, je weet weet je moet volgen.	Procedures geven zekerheid.	procedures /standing orders = zekerheid	standardisatie
Niet meer vertrouwen gekregen, dat het ik daarvoor ook al.	Niet meer vertrouwen gekregen.	geen meer vertrouwen	effect empowerment
Ik vind het positief dat er naar gevraagd wordt, zodat jet het zelf eens kan zeggen.	Positief dat ernaar gevraagd wordt.		gevoel over interview
A1, bachelor, master zijn daar allemaal veel vlugger mee weg. Die hebben meer die info gehad.		diploma invloed op aanpassingsvermogen	aanpassingsvermogen collega's vernieuwingen
Over die vernieuwingen, hoe het moet draaien, het reilen en het zeilen.	A1, bachelor, master zijn vlugger weg met vernieuwingen, hoe het moet draaien, reilen en zeilen. Die hebben meer info gehad in de opleiding.	diploma invloed op aanpassingsvermogen	aanpassingsvermogen collega's vernieuwingen
Ja, maar heb wel de indruk dat dat wordt gedaan bijv de eight hours. Als je die opleiding (bachelor) hebt gehad, heb je een stapje voor, ga je daar nog opener voor staan voor al die vernieuwing, omdat je dat al een stukje in je opleiding gehad hebt.	Een kleine opleiding vanuit het beleid in de vorm van eight hours.	eight hours	projecten

		eight hours: info	communicatie beleid
Oudere werknemers missen meer achtergrond informatie, waardoor het nu iets moeilijker voor hen is. Nu wordt het al ingebakken in de opleiding verpleegkunde.	Oudere werknemers missen meer achtergrond informatie, waardoor het nu iets moeilijker voor hen is. Nu wordt het al ingebakken in de opleiding verpleegkunde.	andere generaties: verschil achtergrondinformatie	aanpassingsvermogen collega's vernieuwingen
De kwaliteit komt ten goede door de kennis, je gaat kritischer naar dingen kijken.	De kwaliteit komt ten goede door de kennis, kritischer naar dingen kijken.	kritischer kijken	effect van empowerment
		door kritischer kijken	kwaliteit van zorg
Ja de gewone zorgtijd. Je bent met die patient als je voor die computer staat, maar het menselijke, het zorgen.	Ja de gewone zorgtijd, het menselijke zorgen.	gemis aan tijd voor relatie patiënt	tijd voor patiënt
Door de dingen die erbij komen, waar dat je mee moet bezig zijn.	Door de dingen die erbij komen, waar dat je mee moet bezig zijn.	gemis aan tijd voor relatie patiënt	tijd voor patiënt
